# Supplementary material for: The interactome of intact mitochondria by cross-linking mass spectrometry provides evidence for coexisting respiratory supercomplexes
Source: Mol Cell Proteomics. 2017 Dec 8;17(2):216–32. doi: 10.1074/mcp.RA117.000470 (PMC5795388; doi:10.1074/mcp.RA117.000470)
Supplement: Supplemental Data [file supp_RA117.000470_133922_0_supp_23978_fzffwf.zip › spectra_annotation/mito_DR_spectra_annotation/71-1-7-1-4-1.pdf]

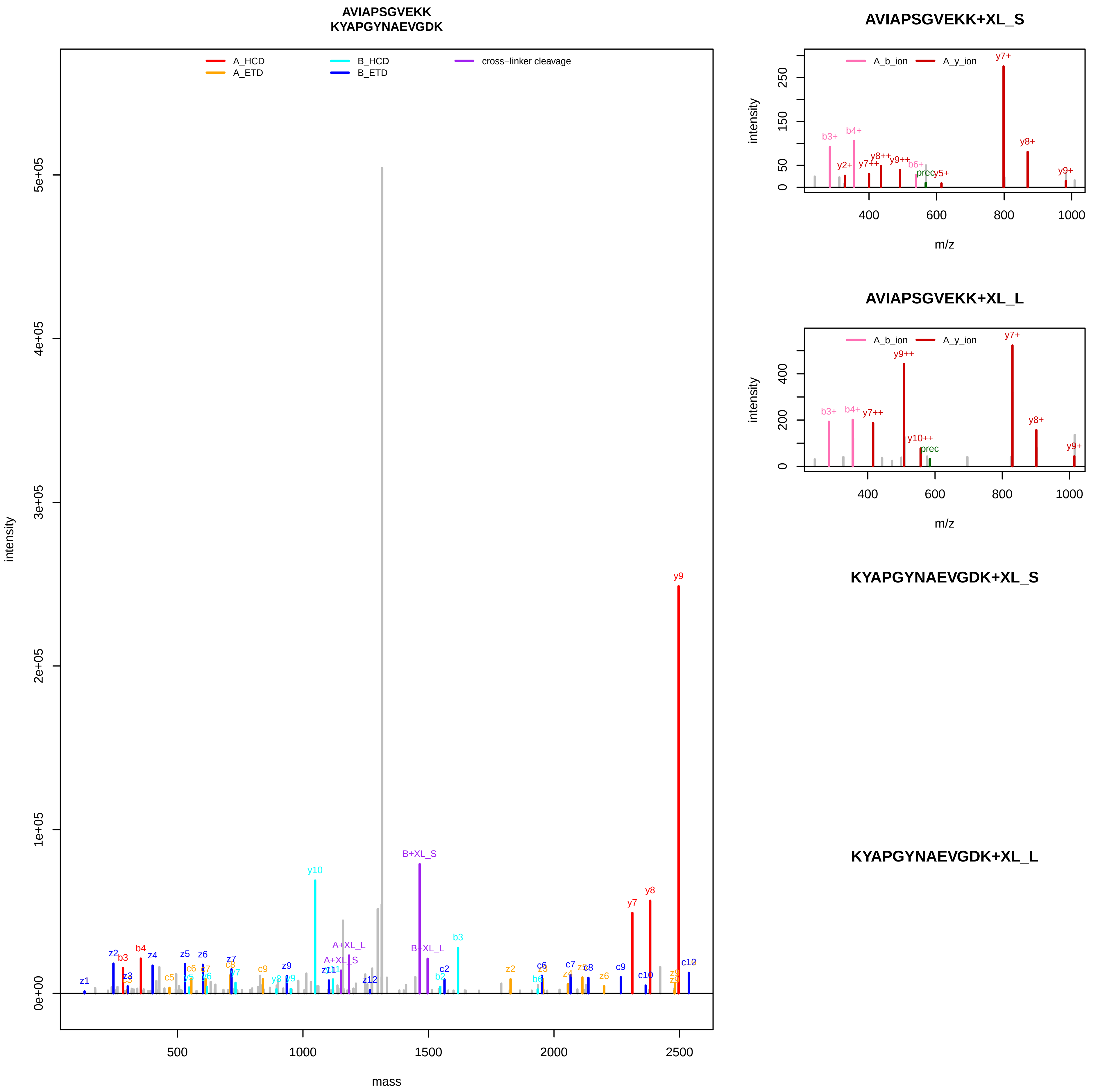

AVIAPSGVEKK
KYAPGYNAEVGDK
A_HCD
A_ETD
B_HCD
B_ETD
cross−linker cleavage
intensity
mass
0e+00
1e+05
2e+05
3e+05
4e+05
5e+05
500
1000
1500
2000
2500
y9
y8
y7
y10
b3
B+XL_S
B+XL_L
A+XL_L
A+XL_S
AVIAPSGVEKK+XL_S
A_b_ion
A_y_ion
m/z
y7+
y8+
y9+
b3+
b4+
b6+
y2+
y5+
y7++
y8++
y9++
prec
AVIAPSGVEKK+XL_L
y10++
KYAPGYNAEVGDK+XL_S
KYAPGYNAEVGDK+XL_L
